# CASE REPORT Complex Wound Closure of Partial Sacrectomy Defect With Human Acellular Dermal Matrix and Bilateral V to Y Gluteal Advancement Flaps in a Pediatric Patient

**Published:** 2013-04-18

**Authors:** J. Bryce Olenczak, Matthew G. Stanwix, Gedge D. Rosson

**Affiliations:** Department of Plastic and Reconstructive Surgery, Johns Hopkins University School of Medicine, Baltimore, Md

## Abstract

**Objective:** Sacrectomy creates a large, complex tissue defect that presents a reconstructive challenge for plastic surgeons. Several myocutaneous flaps have been described for reconstruction following sacral tumor extirpation; however, current publications focus on the reconstructive options applicable to adults. We present a method of reconstruction following sacral tumor extirpation in a pediatric patient. **Methods:** The patient was 22 months old and in need of complex closure following low sacral amputation (S3-S4 osteotomy) and en bloc resection of a yolk sac tumor. Following tumor extirpation, the patient was left with a complex defect including extensive dead space, multiple exposed nerve roots, projection of the rectum into the wound, and inadequate soft tissue for primary closure. **Results:** Reconstruction with human acellular dermal matrix to address the risk of posterior rectal herniation and bilateral gluteal V to Y advancement flaps for obliteration of the dead space allowed for durable closure of the surgical defect. **Conclusions:** This represents the first case report documenting sacral resection and reconstruction with bilateral V to Y gluteal advancement flaps in a pediatric patient.

Reconstruction of the soft-tissue defects resultant from total or partial sacrectomy is described in the adult population; however, discussion of the reconstructive options available to the pediatric population does not exist. Only 1 pediatric case of reconstruction with a myocutaneous flap closure has been reported through 2013.^1^ The 2 most commonly documented options for myocutaneous flap reconstruction of large sacral wound defects include vertical rectus abdominis myocutaneous flaps and bilateral gluteal advancement flaps. Here, we present a pediatric patient with low sacral amputation (S3-S4 osteotomy) and en bloc resection of tumor followed by reconstruction with human acellular dermal matrix (HADM; AlloDerm, LifeCell Corporation, Branchburg, New Jersey) and bilateral V to Y gluteal advancement flaps.

## METHODS/CASE PRESENTATION

An Asian female, age 17 months, was diagnosed with a yolk sac tumor after biopsy of a mass emanating from the sacrum and extending into the buttocks. The patient had a normal birth history, with appropriate achievement of developmental milestones including walking without assistance. She did not appear to have pain related to the mass. Determination of bowel or bladder effects was complicated by her age. She underwent bleomycin chemotherapy and had a good response with a reduction in the size of the tumor. It was felt that she could benefit from surgical resection. Magnetic resonance imaging and computed tomographic scans demonstrated that the tumor was located in the presacral region extending up to the S3 nerve roots and involving the distal sacrum and coccyx.

**Figure 1:**
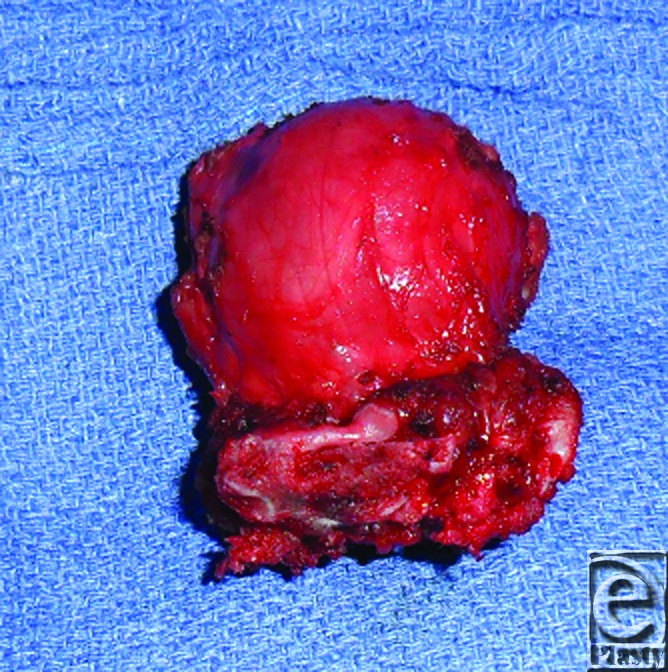
Yolk sac tumor with attached sacral bone measuring 6 cm (length) × 4.5 cm (width) × 4.3 cm (depth).

**Figure 2:**
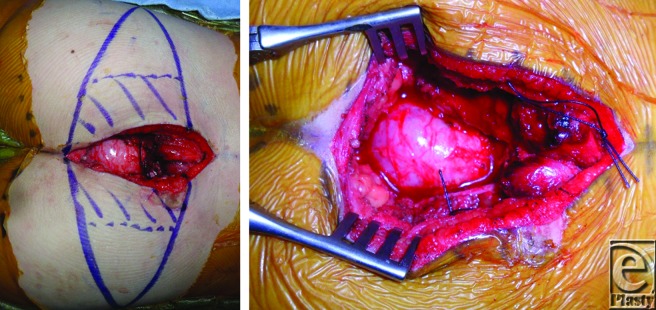
Intraoperative photographs. Patient is positioned prone with head oriented to the right. Hash marks on right buttock indicate area that was de-epithelialized and inset into wound (*left*). Tumor resection resulted in a complex defect with extensive dead space and projection of the rectum into the wound (*right*).

At 22 months of age, the Neurosurgery and Orthopedic Surgery Oncology teams performed low sacral amputation and en bloc resection of the tumor through a posterior approach (Fig 1). The resultant defect was complex: extensive dead space was formed by bony resection, gluteal and other muscles were lateralized by mass effect, multiple exposed nerve roots filled the field, the rectum projected into the wound causing concern for hernia or pseudohernia, and overlying subcutaneous and skin was inadequate (Fig 2).

## RESULTS

**Figure 3:**
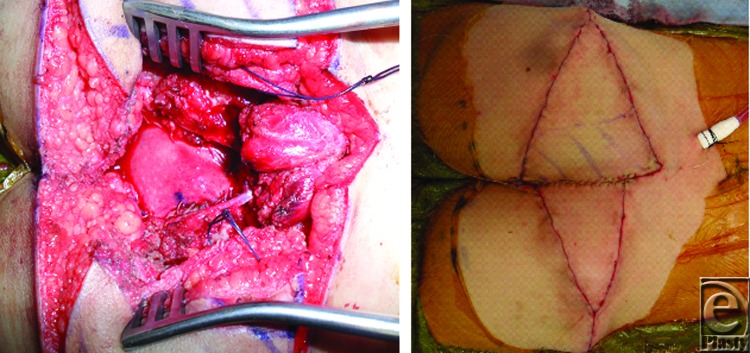
Intraoperative photographs. Patient is positioned prone with head oriented to the right. 4 × 3 cm^2^ piece of HADM was sutured circumferentially to the deep fascia and muscle edges to address posterior rectal herniation (*left*). Closure of the bilateral gluteal flaps in a pants-over-vest fashion with V advancement flaps closed in a Y fashion (*right*). HADM indicates human acellular dermal matrix.

**Figure 4:**
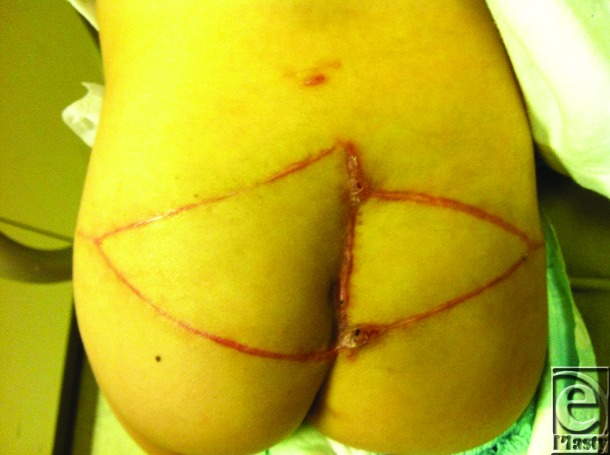
Postoperative photograph after complex wound closure of partial sacrectomy defect at 4 weeks.

The posterior rectal herniation was addressed by suturing a 4 cm by 3 cm piece of HADM circumferentially to the deep fascia and muscle edges surrounding the rectum. Bilateral gluteal V to Y advancement flaps were then designed on each buttock. The flap on the right buttock was partially deepithelialized on the medial aspect, dissected near the gluteal perforators, advanced, and inset deep into the wound to obliterate the dead space. The left buttock flap was then advanced to meet the right flap in a pants-over-vest fashion. The V advancements flaps were closed in a Y fashion (Fig 3). The cumulative surface area of the 2 flaps was 19 cm across by 8 cm in height, representing a significant size in this 11-kg pediatric patient. At the 4 week follow-up, the patient demonstrated durable wound reconstruction without signs of wound infection or dehiscence requiring reoperation (Fig 4).

## DISCUSSION

Resection of sacral tumors by a posterior approach results in large defects that are difficult to reconstruct and present several potential complications including wound dehiscence, impaired motor function, and parasacral herniation. In the adult population, the documented surface area of reconstruction has ranged from 189.8 cm^2^ to 245.7 cm^2^; our patient had a reconstructed surface area of 152 cm^2^.^2^^,^^3^ The defect size relative to patient size created a unique challenge in selecting the appropriate means of closure. In comparison to primary closure, reconstruction with a myocutaneous flap results in a statistically significant reduction in wound infections and dehiscence requiring reoperation.^4^^,^^5^ Several techniques for reconstruction with autologous tissue transfer have been reported including ventral rectus abdominis myocutaneous (VRAM) flaps, omental flaps, gluteal flaps, gluteal-thigh flaps, and free flaps.^1^^-^4^,^^6^^,^^7^

Because our patient required a low sacral amputation, the entire resection was performed by a posterior approach. Therefore, the option of mobilizing a VRAM flap was not considered advantageous because it would result in donor site morbidity in an otherwise nonoperated field. Furthermore, Garvey et al^6^ argue against utilizing a VRAM flap in partial sacrectomies below the S2-S3 level because of the risk of pedicle compression. The bilateral V to Y gluteal advancement flaps have the advantage of large tissue volume, proximity to the defect, and a blood supply that remains intact through the posterior approach.^1^^,^^7^^,^^8^

With respect to the unique complications of reconstruction following partial sacrectomy, there is no statistically significant relationship between flap choice and postoperative complications.^6^ Patient's functional outcome and ambulatory status is primarily dependent on the type of surgical resection rather than on the means of reconstruction.^7^^,^^9^ Although the gluteus maximus is the primary extensor of the hip, hypertrophy of the semitendinosus, semimembranosus, biceps femoris, and adductor magnus compensate in active extension of the hip for activities such as ambulation, climbing stairs, and standing from a seated position.^10^ Finally, inadequate reconstruction following sacral resection may result in parasacral herniation of the rectum.^11^ The sacral defect, soft-tissue dead space, and sacral nerve root resection resulting in pelvic floor atrophy contribute to this problem.^12^^,^^13^ For this reason, we created a pelvic diaphragm using HADM, which is effective in preventing parasacral herniation.^1^

Surgical management of complex sacral wounds in the pediatric patient population following tumor extirpation lacks documented literature. The 22-month-old female underwent resection and partial sacrectomy resulting in a significant defect requiring posterior rectal hernia repair with AlloDerm and complex wound closure with bilateral gluteal V to Y tissue rearrangement. Therefore, this represents the first case report documenting this method of complex sacral reconstruction, with HADM and bilateral V to Y advancement flaps, performed in a pediatric patient.
